# Long-Term Evaluation of Patients Undergoing Genitoplasty due to Disorders of Sex Development: Results from a 14-Year Follow-Up

**DOI:** 10.1155/2013/298015

**Published:** 2013-11-25

**Authors:** Heng Zhang, Jinhong Pan, Huixiang Ji, Yongquan Wang, Wenhao Shen, Limei Liu, Gensheng Lu, Zhansong Zhou

**Affiliations:** ^1^Department of Urology, Southwest Hospital, Third Military Medical University, 30 Gaotanyanzheng Street, Chongqing 400038, China; ^2^Department of Pathology, Southwest Hospital, Third Military Medical University, Chongqing 400038, China

## Abstract

*Purpose*. To summarize the experience in treating patients with genitoplasty due to disorders of sex development in China. *Methods*. The operative procedures, gender of rearing, surgical outcome, and psychosocial and family adjustments of 262 patients were reviewed retrospectively. *Results*. At initial diagnosis, the mean age was 14.3 ± 2.8 years (range: 2–38 years). There were 96 children, 133 adolescents, and 33 adults. Follow-up was done every 6 months. Patients with female sex assignment had no urinary incontinence or voiding difficulty. Five patients underwent the second surgery (3%); vaginal dilation was performed in 35 patients with postoperative vaginal stenosis; 12 patients (7.4%) were unsatisfactory with the outcome. For patients with male sex assignment, the median length of penis was 2.2 cm in prepubertal patients, 4.2 cm in pubertal patients, and 5.0 cm in adults; 39 patients developed postvoid dribbling (39%); 21 patients underwent a second surgery (21%); urethral dilation was done in 28 patients (28%) due to urethral stricture; 38 patients were unsatisfactory with the outcome (38%). In addition, 136 patients (83%) with female sex assignment and 54 (54%) with male sex assignment had favorable psychosocial adjustment. *Conclusions*. Patients with male sex assignment have more surgical complications and difficulties in psychosocial adjustment as compared to those with female sex assignment.

## 1. Introduction

Disorders of sexual development (DSD) refer to congenital conditions with atypical development of chromosomal, gonadal, or anatomical sex [1]. In fertilization, the sex chromosomes of sperm and egg determine the gonad (testes or ovaries) by which sex hormones are secreted. These hormones bind to corresponding receptors determining the development of sexual characteristics. Any abnormality in this process may cause DSD which includes the loss or mutation of SRY gene in chromosome Y resulting in abnormal sexual differentiation and development. Chromosomes X, 11, 17, and 9 are regarded to involve in the regulation of this process [2–6]. In 2006, the European Society for Paediatric Endocrinology (ESPE) and the Lawson Wilkins Paediatric Endocrine Society (LWPES) recommended to describe these diseases as DSD.

 The management of DSD has been a challenge in clinical practice and usually requires multidisciplinary approaches [7]. The most important issue in the treatment of DSD is whether they can achieve the ability to adapt to normal life as males or females in future. However, few studies have been conducted to investigate the long-term physical, psychological, and social outcomes of DSD patients undergoing genitoplasty due to high incidence of loss of follow-up, especially in Asian countries [8]. 

In the present study, the long-term outcomes of DSD patients undergoing genitoplasty were reviewed to evaluate the psychosocial issues, surgical techniques, and sex assignment in the management of DSD. 

## 2. Materials and Methods

### 2.1. Patients

The whole protocol of this study was approved by the Ethics Committee of our hospital. A total of 262 patients undergoing surgical intervention for DSD in our hospital were recruited from 1998 to 2012. At initial diagnosis, the mean age of DSD patients was 14.3 ± 2.8 years (range: 2–38 years). There were 76 males and 186 females before treatment. The patients were classified by karyotype, signs of androgen effects (XY-DSD and XX-DSD as suggested by the consensus statement of ESPE/LWSPE conference) according to methods in study of Lux et al. [9] (Table 1).

1. DSD-XX-P-F: individuals with XX-karyotype and partial androgen effects, reared as girls/living as women (*n* = 49, children: 19; adolescents: 25; adults: 5).
2. DSD-XX-P-M: individuals with XX-karyotype and partial androgen effects, reared as boys/living as males (*n* = 27, children: 8; adolescents: 12; adults: 7).
3. DSD-XX-C-F: individuals with XX-karyotype and without androgen effects, reared as girls/living as women (*n* = 11, children: 4; adolescents: 6; adults: 1).
4. DSD-XY-P-F: individuals with XY-karyotype and partial androgen effects, reared as girls/living as women (*n* = 72, children: 20; adolescents: 44; adults: 8).
5. DSD-XY-C-F: individuals with XY-karyotype and without androgen effects, reared as girls/living as women (*n* = 54, children: 12; adolescents: 34; adults: 8).
6. DSD-XY-P-M: individuals with XY-karyotype and partial androgen effects, reared as boys/living as men (*n* = 49, children: 9; adolescents: 36; adults: 4).

Before surgery, the disease condition and surgical procedures were explained to patients and their relatives. The sex assignment was determined mainly according to the considerations of patients and their relatives, and clinicians provided important advice for them. Moreover, the informed consent was obtained before surgery.

Before surgery, patients were evaluated by endocrinologists and psychologists. Morphology and urination were evaluated by physicians, and the sexual symptoms and satisfaction with surgery were assessed with a questionnaire. 

### 2.2. Diagnosis and Assessment

The diagnostic procedures, surgical intervention, complications, sex assignment, and findings in follow-up were recorded and reviewed retrospectively. The diagnostic procedures included collection of medical history, physical examination, urine and blood steroid measurements, chromosomal analysis, computed tomography (CT) and pelvic ultrasonography, urethroscopy, laparoscopy, and biopsy. The psychosocial adaption assessment in this study was performed according to the described by Köhler et al. and Gupta et al. [10, 11] in which satisfaction with surgery, gender approval, family relationship, social relationship, marriage desire, sexual desire, and work desire were evaluated.

### 2.3. Surgical Procedures

In males, the genitoplasty included urethroplasty, penis straightening, scrotoplasty, and orchidopexy. For patients with severe hypospadia, surgical interventions were performed with an interval of 6 months. Urethroplasty was done with Denis Browne technique or by construction of the urethra with bladder mucosa. When the vagina was evident in the vulva, vaginal resection was done. 

**Figure 1:**
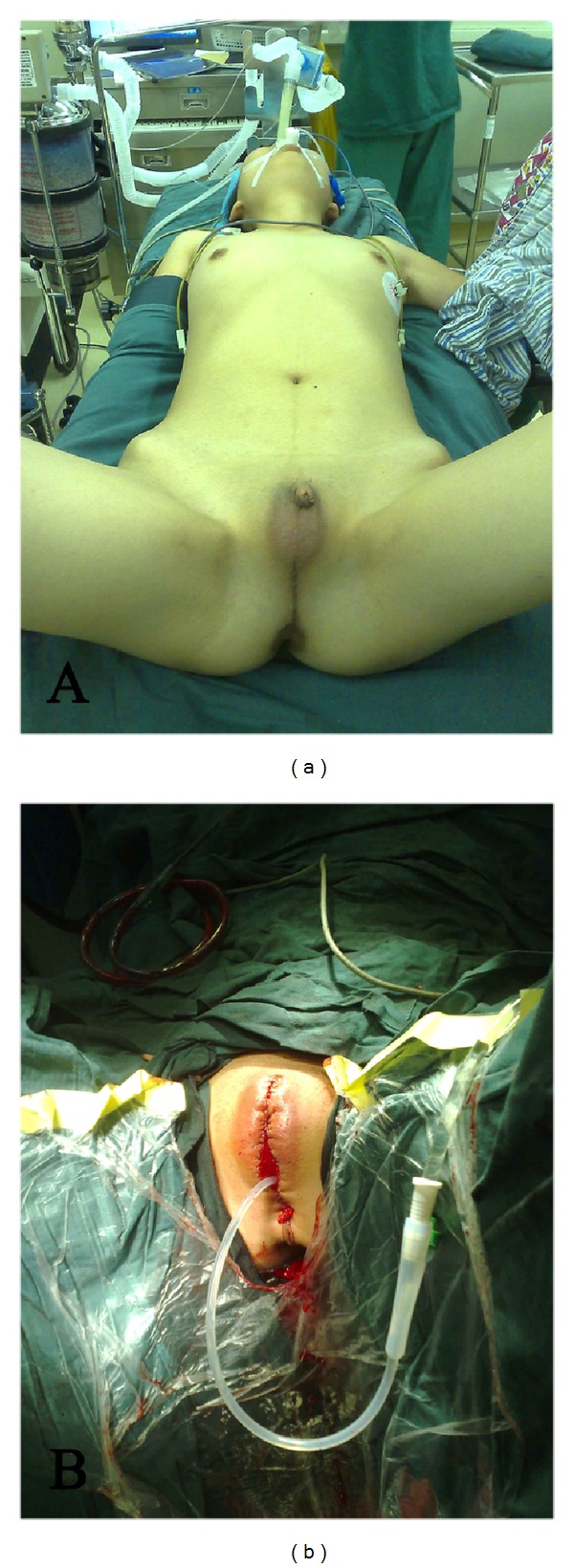
Externalia plasty for ovotesticular DSD. (a) Before surgery. The vagina was not obvious, and the vagina and urethra shared an outlet at the top of small penis with poor development. (b) After surgery. The outlet of the vagina and urethra was exposed, and the vagina and urethra were separated. The urethra was extended for urination control and vagina exposure. Patients agreed with publication of these photos.

For females, the genitoplasty included wedge resection of the clitoris to preserve the vascular and nerve bundles, and plasty of vulvar vestibule, labia minora, and labia majora for normal female appearance. When the vagina and urethra had shared outlet, the vagina and urethra were exposed and separated, the urethra was extended for urination control, and the vagina was exposed (Figure 1). For patients without vagina or with very poorly developed vagina, sigmoid vaginoplasty was performed in the adulthood. In several patients, Williams' vulvovaginoplasty was concomitantly performed during the vulvoplasty in childhood. 

The gonad was preserved or removed depending on the sex assignment. After surgery, hormones were administered. All patients received long-term follow-up and psychological guidance. 

### 2.4. Sex Assignment

The decision making of sex assignment is important in the treatment of DSD. Sex assignment was determined according to patients' considerations, psychological gender, dominant gonad, and development of externalia. The urological surgeon, endocrinologist, and pediatric psychiatrist provided important advice for sex assignment. However, the final decision of sex assignment should be made by the DSD patients and/or their relatives. The Chinese preferring a boy might significantly affect the decision making of sex assignment. 

### 2.5. Follow-Up

DSD patients and their parents were interviewed individually for psychosocial and family adjustments after surgery once every 6 months. DSD patients were guided to correctly look upon the diseases, told some skills to deal with social and family relationships, asked to change their unreasonable mind, emotion, and response, and guided to adapt to the society. For patients with evident psychological disorders, psychologists were invited for interviewing. However, there were still some psychological problems in these patients due to some social factors. During the follow-up period, the treatment outcome, degrees of satisfaction with surgery, social relationship, future expectation, and others were evaluated. 

## 3. Results 

### 3.1. Sex Assignment

The assigned sex was female in 163 patients (62%) and male in 99 (38%). Among DSD-XX-P-F patients, the assigned sex was female in 41 patients and male in 8; among DSD-XX-P-M patients, the assigned sex was female in 2 patients and male in 25; among DSD-XX-C-F patients, the assigned sex was female in 9 patients and male in 2; among DSD-XY-P-F patients, the assigned sex was female in 66 patients and male in 6; among DSD-XY-C-F patients, the assigned sex was female in 43 patients and male in 11; among DSD-XY-P-M patients, the assigned sex was female in 2 patients and male in 47 (Table 1).

### 3.2. Surgical Procedures and Complications

Among 163 patients with assignment of female sex, 25 received only gonad resection because the female externalia was favorably developed. Vulvoplasty was performed in remaining patients, and urinary incontinence and voiding difficulty were not found after surgery. In addition, 5 patients received a second surgery (3%); urethral-vaginal fistula was found in 1 patient; clitoral enlargement was present in 4 patients due to irregular treatment with hormones. For 29 patients with poor vagina development, vaginal dilation was performed at designed time points, but surgery was not performed. For the first 6 patients, Williams' vulvovaginoplasty was concomitantly performed during the vulvoplasty (*n* = 9), but vulval stenosis was observed in 6 patients, and thus vaginal dilation was performed. For patients without vagina or with poorly developed vagina, vaginal dilation usually has unfavorable therapeutic efficacy, and the sexual intercourse in adulthood is often infeasible. Thus, sigmoid vaginoplasty was done in most of these patients (*n* = 65). For patients with assignment of male sex, the median length of penis was 2.2 cm in children, 4.2 cm in adolescents, and 5.0 cm in adults after surgery, and the outlet of the urethra was found in the glans penis. In addition, 39 patients complained of poor urine flow rate and postvoid dribbling (39%); 21 received a second surgery due to complications (21%) of whom penis augmentation was done in 8; 6 received surgery for urinary fistula; 7 underwent surgical intervention due to urethral stricture and difficult urination; urethral dilation was done at designed time points in 28 patients; 1 developed a gonadal tumor (Table 2).

### 3.3. Age of Patients Undergoing Surgery

Among 96 children receiving surgery, the median age was 6.4 years; of 133 adolescents undergoing surgery, the median age was 14.2 years; among 33 adults, the median age was 23.2 years. In addition, 99 of 133 adolescents (74%) and 26 of 33 adults (78%) thought that it was late to receive surgical intervention. 

### 3.4. Psychosocial Adaption

A patient with DSD-XY-P-M and physiological gender of female did not approbate the gender, and a second sex assignment surgery was performed. The remaining patients approbated the gender. Among patients with the assignment of female sex, 12 (7%) were unsatisfactory with outcome due to disparity with real genitalia in appearance. Among patients with assignment of male sex, 38 were unsatisfactory with outcome, and they usually worried about small penis (*n* = 35; 35%) and less semen (*n* = 18; 18%). Good social relationship was found in 136 females (83%) and 54 males (54%). Good family relationship was noted in 153 females (94%) and 72 males (73%). Among 51 adult females (>20 years), 42 had sexual desire (82%) of whom 32 were satisfactory with outcome (76%), 41 had marriage desire (80%) (12 were married and 19 expected marriage), and 46 had work desire. Among 39 adult males, 15 had sexual desire (38%) of whom 9 were satisfactory with outcome (60%), 13 had marriage desire (33%) (5 were married and 8 expected marriage), and 22 had work desire (Table 2). 

## 4. Discussion

Patients with DSD and their family members usually experience great pain, and thus it is important to develop strategies for the prevention and treatment of DSD. The goal of DSD treatment is to correct sex disorders and improve social and psychological conditions, in which surgery, endocrine therapy, and psychological therapy are often applied. These therapies attempt to adapt patients to future life and social activities. However, DSD treatment is still a challenge to neonatologists, pediatric endocrinologists, and pediatric surgeons [12]. To date, although some studies have been conducted to investigate the etiology and treatment of DSD, studies with large sample size and long-term follow-up are less reported [13], especially in Asian countries. In addition, DSD treatment usually depends on experience and is often not supported by the evidence-based medicine. Thus, single centered study with large sample size and long-term follow-up may provide important evidence for clinical practice. In the present study, DSD patients with surgical intervention in our hospital were retrospectively reviewed. 

**Figure 2:**
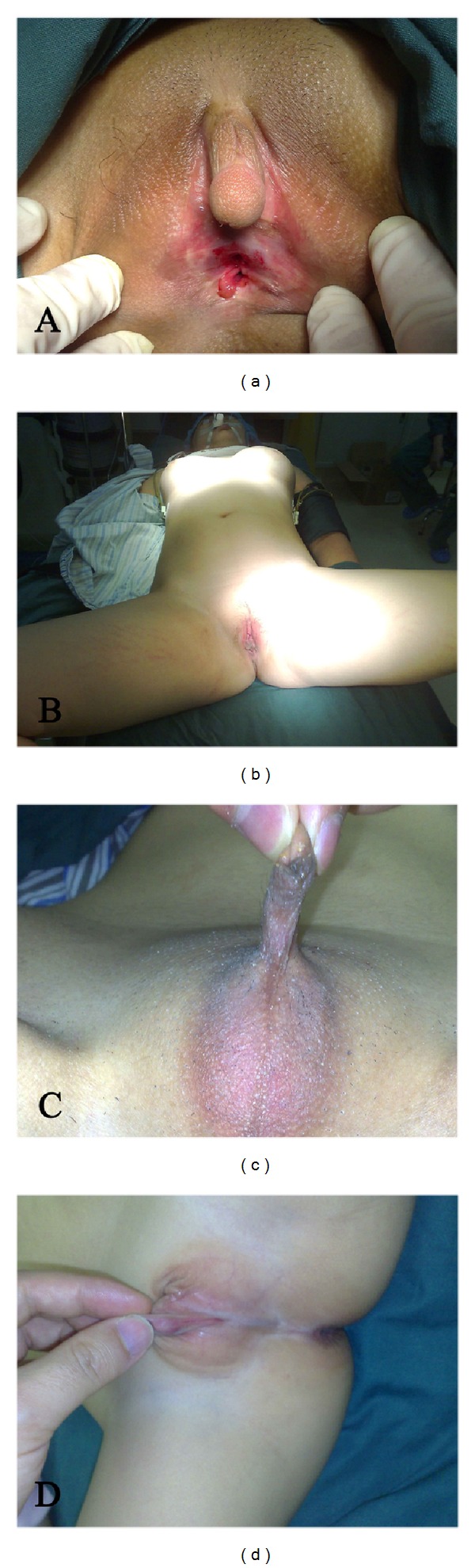
DSD patients. (a) 46, XX DSD. The clitoris was hypertrophic and penis-like, the gonad was ovoid, and the patient had naïve uterus; (b) 46, XY DSD. Complete female externalia was observed in appearance, the vagina and breast were well developed, and bilateral testicles were found in the abdomen. (c) Ovotesticular DSD. The penis was poorly developed, and unilateral testicle and contralateral ovary were found; (d) 46, XY. Complete gonadal dysgenesis was observed. The vagina and urethra shared an outlet and the gonads were poorly developed. Patients agreed with publication of these photos.

Before 2008, term hermaphroditism was used and can be clinically classified into female pseudohermaphroditism, male pseudohermaphroditism, true hermaphroditism, and gonadal dysgenesis. Currently, new classifications are employed for medical recording and data analysis. The 46, XX DSD is usually caused by 21- or 11-hydroxylase deficiency or congenital adrenal hyperplasia; the 46, XY DSD is often attributed to the secretion of androgen and abnormalities in metabolism and/or receptors. Our results showed that 46, XY DSD was the most common (67%) and most complex DSD and could present with whole female genitalia in appearance (Figure 2(b)). Ovotesticular DSD manifests the presence of both ovaries and testicles, but it has a low incidence. In the present study, about 7% of patients were diagnosed with ovotesticular DSD. 

For patients with DSD, the gender of rearing, psychological status, and family history should be recorded in detail on admission, and physical examination be performed to confirm the development of externalia. In addition, examinations of karyotype and sex hormone and imaging examination are required. Ureteroscopy is helpful to identify the conditions of the vagina and urethra, and laparoscopy is beneficial to examine the gonad and uterus and for the biopsy and subsequent surgery [14]. We proposed that urethroplasty and phalloplasty should be performed in an early phase for patients with assignment of male sex, but vaginoplasty should be done after adulthood for patients with assignment of female sex, which may reduce complications. Early diagnosis and disclosure of the diagnosis, counseling of patients and families, and social support were also done in our country.

Not all DSD children are treated at infant stage. The clinical manifestations of DSD vary among patients, and thus it is difficult to confirm the diagnosis of DSD and to predict the future sexual development. In particular, the etiology of 46XY DSD is complicated, and the clinical presentations of 46XY DSD are also diverse. Some clinicians propose that for children with difficulty in predicting penile development, the gender of rearing is determined on the basis of sexual characteristics, and corresponding surgical intervention is then applied. However, DSD is not accepted by the society, and patents are impatient to wait for surgical intervention. In addition, with the changes in society and social perception, only the gender of rearing is accepted by the society, and patients and their family members emphasize family factors, social background, and psychological factors. Nevertheless, clinicians usually pay attention to physiological gender because it is related to therapeutic efficacy and quality of life. Thus, clinicians should explain the disease conditions to patients' relatives, communicate with their patients, and provide information on DSD treatment for their patents and relatives in advance. Patients, patients' relatives, and clinicians may cooperate to make decision. The age at diagnosis is crucial for decision making. The older the patients are, the more they are susceptible to influence of social and psychological factors. In particular, in developing countries in Asia, the economy and culture are at low levels, and the diagnosis is usually performed at late stage of diseases. Moreover, some DSD children often became self-abased and introverted and communicate less with outside world. Thus, the psychological problems should be emphasized in these patients. However, there is still a big gap in social adaption and psychological well-being of patients between developing countries and developed countries. The psychological evaluation is difficult and patients are susceptible to loss of follow-up in developing countries. Thus, in the present study, only basic psychosocial evaluation was done to investigate the current status of DSD treatment and the influence of social and culture factors on DSD treatment. However, absence of standard and comprehensive evaluation of psychosocial adaption is a limitation of this study. With the accumulation of cases, we will pay more attention to the psychosocial adaption in these patients. In addition, there are a large number of DSD patients in China, which allows the investigation of DSD in a relatively large population. 

The decision making of sex assignment is critical for DSD treatment. Previously, the sex assignment was determined according to the degree of externalia masculinization, length of penis, patients' growth, and future fertility, but the chromosome condition is not used as a factor for sex assignment [15]. However, Yassin proposed that the sexual orientation has been determined in the embryonic phase or early after birth [16]. Thus, the decision making on sex assignment should be based on the psychological gender, development of externalia and dominant gonad, as well as on the considerations of patients and their family members [17, 18]. Early diagnosis and early treatment are beneficial for early establishment of normal gender and psychology [19]. However, this is still a challenge. Whether patients adapt to the society in the assigned gender is still unclear, especially, for patients with assignment of male sex. Kojima et al. investigated 12 patients with assignment of male sex. Their results showed that although DSD males had an undeveloped penis and testis and hypergonadotropic hypogonadism or normogonadism, most of them had male sexual potential and male sex identity as long as testicular tissues were preserved [20]. Gupta et al. found that despite parents provided moral, social, and economic support, DSD children continued to have apprehensions in social adjustments. In the present study, 62% of males and 83% of females were satisfactory with outcome; good social adjustment was found in 54% of males and 83% of females. In addition, 38% of males and 82% of females had sexual desire and 33% of males and 80% of females had marriage desire. The assignment of male sex predicted a greater burden on physical and mental health and social adjustment. In China, the sex assignment is frequently influenced by Chinese culture. As in other developing countries in Asia, more family members prefer assignment of male sex. Thus, clinicians should carefully provide advice for patients on the basis of psychological status, gender of rearing, and difficulty in anaplasty of external genitalia.

Once sex assignment is completed, the externalia malformation should be treated. For patients with assignment of female sex, wedge resection of clitoris with preservation of neurovascular bundle is recommended [21], but hormone treatment should be maintained to control the androgen level. In the present study, 4 patients had poor compliance to hormone treatment, resulting in clitoral enlargement which required a second surgical intervention. For children, hormone is often administered to promote vagina development. When they are in adulthood or in need of sexualization, vaginal dilation or sigmoid vaginoplasty can be performed, and complications (such as vaginal stenosis) are seldom noted [22]. For patients with assignment of male sex, the surgical procedures are more complex, and the success rate of surgery is at a low level. Surgery should be performed in early phase to assure favorable outcome. Generally, penis straightening, urethroplasty, scrotoplasty, and testicle fixation are applied. In our hospital, urethroplasty is usually performed with Denis Browne technique or done with bladder mucosa. The common complications were urinary fistula (6%) and urethral stricture (7%), and the majority of patients were unsatisfactory with the size of penis (35%), which may affect the physical and mental health and social adjustment in these patients. Symptoms related to voiding were absent in patients with assignment of female sex, but 39% of patients with assignment of male sex developed postvoiding dribbling. Thus, patients with assignment of male sex have higher risk for surgical complications. In addition, the surgery is easier for patients with assignment of female sex. Our results showed that 21% of males and 3% of females received a second surgery due to complications. Currently, the biological and tissue engineering materials (penile/testicular prosthesis) may be beneficial for surgical intervention in patients with assignment of male sex [23, 24]. 

The hypogenetic gonad has a high risk for canceration, and thus these patients should be monitored closely even the testis falls into the scrotum [25]. However, there is controversy on the timing of hypogenetic gonad resection. Some surgeons propose that resection of hypogenetic testicles should be performed as early as possible due to its cancerogenetic potential. However, other clinicians propose that application of orchiectomy should be careful, and orchiectomy should be performed only when the hypogenetic gonad presents with cancerogenetic tendency or the gender of rearing has been determined because the testis is critical for not only physical but also psychosocial maturation in these children [26]. In our experience, for XY DSD male patients with difficulty in predicting prognosis, primary surgery is recommended to fall the testis to the inguinal region, and further treatment is determined according to the etiology of disease and development of patients. For patients with underdevelopment and gender nonconformity, additional hormone is required. However, these procedures should be accepted by their parents. In our previous study, the pelvic gonad in 1 patient with DSD-XY-C-F progressed into mixed germ cell tumor [27].

The goal of DSD treatment is to help patients to adapt to the society. DSD is a special disease and is frequently found in rural areas of China. Some patients are unwilling to see a doctor due to economic and cultural concerns. Thus, the diagnosis of DSD in these patients is usually done at late stage. Under this condition, DSD treatment is difficult. Moreover, DSD patients usually have some psychological problems in social adjustments. Thus, close follow-up and regular psychological guidance should be provided on the marriage, giving birth, interpersonal relation, and employment. With the development in China, this condition is improved significantly. Decision making of sex assignment is a complex physiological, psychological, and social issue. Chinese preferring a boy may affect the decision making of sex assignment, and clinicians can provide advice for patients and their family members. However, the decision of sex assignment is finally made according to considerations of patients and their relatives. 

## Figures and Tables

**Table 1:** Distribution of etiologies in sex assignment of female and male groups.

	Diseases					
	DSD-XX-P-F	DSD-XX-P-M	DSD-XX-C-F	DSD-XY-P-F	DSD-XY-C-F	DSD-XY-P-M
	(*n* = 49)	(*n* = 27)	(*n* = 11)	(*n* = 72)	(*n* = 54)	(*n* = 49)
Female sex assignment (*n* = 163)	41	2	9	66	43	2
Male sex assignment (*n* = 99)	8	25	2	6	11	47

**Table 2:** Surgical outcome and psychoscial adaption in sex assignment of female and male groups.

	Second surgery	Satisfaction with surgery	Gender approval	Good family relationship	Good social relationship	Sexual desire	Satisfaction with sexualization	Marriage desire	Work desire
		(Y/N)	(Y/N)	(Y/N)	(Y/N)	(Y/N)	(Y/N)	(Y/N)	(Y/N)
Female sex assignment (*n* = 163, adults: 51)	5	151/12	162/1	153/10	136/27	42/9	32/19	41/10	46/5
Male sex assignment (*n* = 99, adults: 39)	21	61/38	99/0	72/27	54/45	15/24	9/30	13/26	22/17
